# Fabrication and Plasma Surface Activation of Aligned Electrospun PLGA Fiber Fleeces with Improved Adhesion and Infiltration of Amniotic Epithelial Stem Cells Maintaining their Teno-inductive Potential

**DOI:** 10.3390/molecules25143176

**Published:** 2020-07-11

**Authors:** Mohammad El Khatib, Annunziata Mauro, Ralf Wyrwa, Miriam Di Mattia, Maura Turriani, Oriana Di Giacinto, Björn Kretzschmar, Thomas Seemann, Luca Valbonetti, Paolo Berardinelli, Matthias Schnabelrauch, Barbara Barboni, Valentina Russo

**Affiliations:** 1 Unit of Basic and Applied Biosciences, Faculty of Bioscience and Agro-Food and Environmental Technology, University of Teramo, 64100 Teramo, Italy; melkhatib@unite.it (M.E.K.); mdimattia@unite.it (M.D.M.); mturriani@unite.it (M.T.); odigiacinto@unite.it (O.D.G.); lvalbonetti@unite.it (L.V.); pberardinelli@unite.it (P.B.); bbarboni@unite.it (B.B.); vrusso@unite.it (V.R.); 2 Department of Biomaterials, INNOVENT e. V., 07745 Jena, Germany; rw1@innovent-jena.de (R.W.); ms@innovent-jena.de (M.S.); 3 Department of Surface Engineering, INNOVENT e. V., 07745 Jena, Germany; bk@innovent-jena.de (B.K.); ts2@innovent-jena.de (T.S.)

**Keywords:** aligned microfibers, amniotic epithelial stem cells, cold atmospheric plasma, electrospinning, exposure time, PLGA, tendon tissue engineering, working distance

## Abstract

Electrospun PLGA microfibers with adequate intrinsic physical features (fiber alignment and diameter) have been shown to boost teno-differentiation and may represent a promising solution for tendon tissue engineering. However, the hydrophobic properties of PLGA may be adjusted through specific treatments to improve cell biodisponibility. In this study, electrospun PLGA with highly aligned microfibers were cold atmospheric plasma (CAP)-treated by varying the treatment exposure time (30, 60, and 90 s) and the working distance (1.3 and 1.7 cm) and characterized by their physicochemical, mechanical and bioactive properties on ovine amniotic epithelial cells (oAECs). CAP improved the hydrophilic properties of the treated materials due to the incorporation of new oxygen polar functionalities on the microfibers’ surface especially when increasing treatment exposure time and lowering working distance. The mechanical properties, though, were affected by the treatment exposure time where the optimum performance was obtained after 60 s. Furthermore, CAP treatment did not alter oAECs’ biocompatibility and improved cell adhesion and infiltration onto the microfibers especially those treated from a distance of 1.3 cm. Moreover, teno-inductive potential of highly aligned PLGA electrospun microfibers was maintained. Indeed, cells cultured onto the untreated and CAP treated microfibers differentiated towards the tenogenic lineage expressing tenomodulin, a mature tendon marker, in their cytoplasm. In conclusion, CAP treatment on PLGA microfibers conducted at 1.3 cm working distance represent the optimum conditions to activate PLGA surface by improving their hydrophilicity and cell bio-responsiveness. Since for tendon tissue engineering purposes, both high cell adhesion and mechanical parameters are crucial, PLGA treated for 60 s at 1.3 cm was identified as the optimal construct.

## 1. Introduction

Tendon disorders, including tendinopathies, represent a severe pathology due to their high incidence rate (about 30 million musculoskeletal lesions/ year), together with a prevalence estimated to increase yearly worldwide [1,2,3]. The conventional treatments used for tendinopathies, including auto-, allo- and xenografts, have shown limited successes so far [2,4,5,6]. Alternative therapeutic strategies can be found in the field of tissue engineering in which biomimetic materials may be engineered with cells to provide a feasible microenvironment to treat tendon disorders [2,7].

Scaffolds offer intrinsic properties to sustain the growth of the cells, their adhesion, spread, proliferation and differentiation and hence facilitate new tissue formation [8,9]. Polymers belonging to the polyester family such as poly(L-lactide) (PLLA), poly(lactide-co-glycolide) (PLGA), and poly-ε-caprolactone (PCL) have been widely used in tissue engineering due to their controlled biodegradability profile, considered a crucial parameter during tendon regeneration [7].

Electrospinning, one of the most suitable and promising techniques available to produce scaffolds for tissue engineering applications [10,11,12,13,14], aims at obtaining fibrous matrices with biomimetic micro- to nanoscale diameters resembling the architecture of the native extracellular matrix (ECM) [14,15,16]. Scaffolds with adjustable geometry, surface chemistry, and mechanical properties can be obtained by controlling the electrospinning process parameters making it a versatile technique in this field [17,18,19,20]. For tendon tissue engineering, in comparison with the other conventional techniques, electrospinning has the advantage of producing scaffolds that mimic the native tendon ECM characterized by aligned collagen fibers [21,22,23] providing topographical cues that promote cell tenogenic differentiation improving subsequently the potential of regeneration [10,11,12,24,25,26,27,28].

Once the topographical properties of electrospun fibers in terms of ECM mimicry are well defined, a biofunctionalization step must be undertaken. This step aims at facilitating cell affinity towards the fibers by promoting the recognition of bound growth factors and specific proteins by cell receptors [29,30]. Many authors have chosen to functionalize the electrospun scaffolds and modify their surfaces by blending the polymer solution with bioactive molecules prior to electrospinning [19,31,32,33,34]. Others have used a wet chemistry surface modification approach to functionalize electrospun scaffolds by immersing them into harsh chemicals such as strong acids or alkalis [35,36,37,38,39,40] in which hydroxyl or carboxyl groups are formed by hydrolyses of ester bonds. Although this treatment has shown to improve hydrophilic properties of the scaffolds with an improvement in cell adhesion and spreading [35,37,38,39,40], treated materials exhibited a strong shrinkage and the fibers lost their alignment with a decrease in the Young’s modulus properties by using a high NaOH concentration [38]. This type of surface modification alters the bulk physical properties of the electrospun scaffolds due to the scission of the polymer backbone due to the harsh processing conditions [35,37,38,39,40,41].

Non-thermal plasma (NTP) treatment can modify the surface properties of polymers to a depth of 10 nm without affecting its bulk properties [42]. It is considered as a greener alternative treatment since it is performed in the gaseous phase without requiring any harsh chemicals. The advantages of this technique rely on the possibility to control and tune all process parameters avoiding in turn nanofibrous structure damages [29]. Different working gases such as air, oxygen (O_2_), nitrogen (N_2_), ammonium (NH_3_), argon (Ar), or helium (He) have been used for this purpose [29,43,44,45,46,47,48]. Most of studies concerning plasma activation of electrospun scaffolds have focused on PCL [29,46,47,49,50,51] and PLLA [52,53,54,55,56,57,58] while those concerning PLGA [48,59,60,61,62] are few and still neglected in the literature although its wide application range in the field of tissue engineering [63]. Despite the known cytocompatibility of PLGA [10,11,13,25,64], its poor hydrophilic properties and the rather low ability to interact with cells restrict the natural cell recognition sites on its surface, which may lead to poor overall cell adhesion [65]. For this reason, surface treatments become a necessity to modify the surface properties of electrospun PLGA scaffolds to improve whether their hydrophilic properties and cell adhesion features. Considering that PLGA is a heat-sensitive material [66], cold atmospheric plasma (CAP) represents a suitable technique for surface modifications due to its operability at room temperature or slightly above this temperature, under atmospheric conditions. In contrast to low pressure or high vacuum plasma techniques that require the use of vacuum chambers, portable devices can be employed to generate CAP making its application desirable for industrial purposes [42]. It has been demonstrated that the plasma treated PLGA materials using different working gases have shown an increase in their hydrophilic properties improving fibroblast [67], rat bone marrow mesenchymal stem cells [45], mouse myoblasts [68], nerve cells [62,69] adhesion, and proliferation due to introduction of specific functional groups.

To our knowledge, no studies have been conducted yet assessing the effect of plasma treated electrospun scaffolds on improving stem cells performance used for tendon tissue engineering. Despite the wide range of mesenchymal stem cells used in tendon tissue engineering [70,71,72,73,74,75,76,77,78,79], amniotic epithelial stem cells (AECs) represent a promising alternative cell source due to their high plasticity [80,81,82] and high immunomodulatory properties [83,84,85,86,87,88]. Moreover, the ability of AECs to differentiate towards the tenogenic lineage has been demonstrated in-vitro [88] and in-vivo exhibiting a high regenerative potential [84,89,90,91,92].

In particular, it has been specifically demonstrated on ovine AECs (oAECs) that electrospun PLGA fleeces with highly aligned fibers and a fiber diameter size in the micron range (i.e., 1.27 µm) induced a tenogenic differentiation after only 24 h of culture, without any supplementation of tenogenic differentiative media [10]. In addition, these topographical cues have shown to increase also oAECs’ immunomodulatory properties [25]. Despite these interesting results, surface treatments could give the advantage to modify the hydrophobic properties of the PLGA copolymer, by changing their surface properties to improve their wettability and cell adhesion property so that they can be used for various applications without altering their mechanical and teno-inductive potential.

For this purpose, the CAP treatment, which is known to increase the hydrophilicity of rather hydrophobic scaffold materials like polycaprolactones, polylactides and their copolymers, was used in this study to improve cell adhesion. Moreover, the use of this well-known technique for activation of electrospun fibers is currently described in only relatively few publications [93,94,95,96] and requires a comprehensive investigation and optimal adjustment of the plasma treatment parameters to maintain the mechanical and teno-inductive integrity. Nitrogen (N_2_) plasma could represent an ideal working gas due to its less aggressivity and destructivity compared to oxidizing plasma (O_2_) that lead to surface etching and destruction due to its high molecular size [97,98].

Based on these premises, this research was designed to assess the effect of CAP using N_2_ gas on electrospun PLGA fleeces with aligned fibers to modify their surface characteristics. The obtained fleeces were characterized for their physicochemical and mechanical properties and they were tested for their bioactivity on oAECs evaluating cell adhesion, penetration, proliferation rate, and teno-differentiation.

## 2. Materials and Methods

### 2.1. Materials

Poly(lactide-co-glycolide) (PLGA, PLG8523) with an inherent viscosity midpoint of 2.3 dL·g^−1^ and a lactide to glycolide ratio of 85:15 was purchased from PURASORB^®^ (Corbion Purac, Gorinchem, The Netherlands). The determination of molecular weight (M_w_ = 258,000 g·mol^−1^) has been done using gel permeation chromatography (GPC) by dissolving the polymer in chloroform and using polystyrene as external standard. Hexafluoro-2-propanol (HFIP) (99%) was obtained from Apollo Scientific (Manchester, UK). Chloroform used as eluent for GPC analysis was purchased from Fisher Scientific (Schwerte, Germany). All other chemicals and solvent were of analytical grade and used as received.

### 2.2. Fabrication of the Aligned PLGA Microfiber by Electrospinning

**Figure 1:**
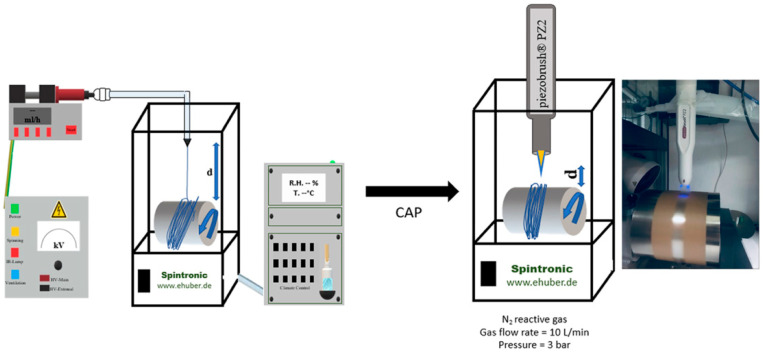
Experimental design of electrospun PLGA microfibers fabrication and Cold Atmospheric Plasma (CAP) performance. (“d” is the distance between the needle and the collector in the electrospinning process machine (left) and the distance between the plasma handled device and the collector during the plasma treatment process (right)).

A commercial E-Spintronic electrospinning apparatus (Erich Huber, Gerlinden, Germany) with a controllable climate chamber is employed to electrospun the PLGA microfibers. During the process (Figure 1), PLGA solution (12% wt/wt) dissolved in HFIP is loaded into a polypropylene syringe of 5 mL (inner diameter = 0.8 mm) and pumped through a 35 cm PTFE tube (Intra Special Catheters, Rehlingen-Siersburg, Germany). Once the solution droplet comes out of the needle tip, a voltage of 33 kV is applied to the collecting target using a power supply and the flow rate was set at 0.25 mL·h^−1^ and controlled through the syringe pump. The environmental conditions were optimized and set at 22.5 °C and 65% for temperature and relative humidity, respectively. The microfibers were collected on baking paper placed on the metallic cylindrical drum rotating collector (diameter = 12 cm and circumference = 38 cm) located at a distance of 20 cm from the syringe tip. The rotational speed of the collector was optimized to 1000 rpm to obtain fibers with highly aligned topography. The fabricated PLGA microfibers were obtained by electrospinning 250 µL of PLGA solution. The microfibers matrices were vacuum dried for 24 h at room temperature to eliminate solvent residue before being used for further investigations.

### 2.3. Plasma Treatment

The handled device piezobrush^®^ PZ2 (Relyon Plasma, Regensburg, Germany) is used to generate plasma and treat the surfaces of PLGA microfibers. This device uses the so-called piezoelectric direct discharge (PDD) technology which is based on the direct electrical discharge at an open piezoelectric transformer. By the transformed input voltage, a high electric field strength is created that in turn dissociates and ionizes the surrounding process gas. The device was fixed using two clamps as shown in Figure 1. In order to evaluate the effect of working distance and exposure time, the PLGA microfibers were placed beneath the fixed plasma device at two different working distances 1.3 and 1.7 cm and exposed to nitrogen (N_2_) generated plasma for three different exposure times (30, 60, and 90 s) at a gas flow rate of 10 L·min^−1^ and a pressure of about 3 bars. These values were considered according to preliminary experiments where it was found that at long exposure time and a working distance lower than 1.3 cm from the PLGA microfibers showed severe damages (morphological and dimensional alterations) (data not shown). In the text, the distances 1.3 and 1.7 cm were coded as A and B, respectively. The different treated groups were nominated as PLGA followed by the treatment exposure time (30, 60 or 90) and the letter A or B to specify the distance from which the treatment has been conducted (Table 1). Untreated PLGA microfibers, noted PLGA, were used as control.

### 2.4. SEM Analysis

The surfaces of the untreated (PLGA) and plasma treated PLGA microfibers have been examined and imaged using a Supra 55VP field-emission SEM at 5 kV accelerating voltage (Carl Zeiss AG, Jena, Germany) after being gold coated using a sputter coater. Using the imageJ software (NIH, Wisconsin, WI, USA), the average fiber diameter size was determined from around 100 fibers from each fleece type randomly chosen (*n* = 3 for each fleece type) while the changes in fiber orientation before and after CAP treatment were assessed using the directionality Plugin (*n* = 3 for each fleece type). This plugin chops the image into square pieces and computes their Fourier power spectra allowing the generation of statistics data on the basis of the highest peak found represented by direction (the center of the Gaussian), dispersion (the standard deviation of the Gaussian), and goodness (the goodness of the fit, 1 is good and 0 is bad).

### 2.5. Physicochemical Characterization of the PLGA Surfaces

#### 2.5.1. Fourier Transform Infrared Spectroscopy

The untreated (PLGA) and CAP treated PLGA microfibers (*n* = 3 for each fleece type) were analyzed by Fourier transform infrared spectroscopy (FTIR) using an Nicolet iS10 FTIR spectrometer (Thermo Fisher Scientific, S.p.A., Milan, Italy) using an average of 64 accumulations and a resolution of 4 cm^−1^ in the range of 4000–650 cm^−1^. Three samples with the same conditions were used in this analysis.

#### 2.5.2. X-ray Photoelectron Spectroscopy (XPS)

The elemental chemical surface composition and chemical binding properties of the untreated and plasma treated PLGA microfibers were assessed by XPS (AXIS ULTRA spectrometer, Kratos, Manchester, UK) as previously described in [99]. Briefly, a monochromatic Al Kα line (E 1486 eV, 150 W), implemented charge neutralizer, and pass energy of 80 and 10 eV were used to determine the chemical elemental composition of the samples and the highly resolved C1 peaks using the recorded spectra. Three XPS measuring steps from 3 different samples treated with the same conditions were used to determine the average of each surface composition value.

#### 2.5.3. Water Contact Angle (WCA)

To get insights on the surface wettability of the materials, the water contact angles (WCA) of the untreated (PLGA) and CAP treated PLGA microfibers were analyzed using the contact angle measurement system OCA 15 (Data Physics Instruments, Filderstadt, Germany). A distilled water drop (1 µL) is deposited on the surface of PLGA microfibers after which an immediate determination of the drop profile is performed using Young-Laplace-fit method (SCA20 software, V.4.5.11). The average of WCA was calculated based on five independent determinations at different sites of three samples treated under the same conditions conducted at room temperature.

#### 2.5.4. Gel Permeation Chromatography (GPC)

Gel Permeation Chromatography (GPC) investigations were conducted on the (PLGA) and CAP treated PLGA microfibers (*n* = 3 for each fleece type) using a Shimadzu system (Shimadzu Deutschland, Duisburg, Germany). A PSS-SDV (100 Å, 8 × 50 mm) pre-column and a PSS-SDV (100 Å, 8 × 300 mm) column were used for the separation. Weighed samples were dissolved in mobile phase of chloroform (CHCl_3_, stabilized with 1% amylene) at a concentration of 5 mL·h^−1^. The analyses were conducted at 25 °C. The eluent was delivered at a flow rate of 1 mL·min^−1^ and the injection volume was set at 100 µL. A refractive index detector an RID 10A (Shimadzu Deutschland) was applied. Polystyrene standard samples (PSS-Polymer Standards Service, Mainz, Germany) were used for calibration.

### 2.6. Assessment of Mechamical Properties of the Untreated and CAP Treated PLGA Fleeces

The untreated and CAP treated PLGA microfibers were assessed for their mechanical properties with stress-strain analysis conducted at room temperature using a Texture Analyzer TA.XT2i (Stable Micro Systems, Godalming, UK) with a 5 kg load cell. Rectangular pieces of each PLGA fleece group have been prepared with dimensions of 50 mm × 5 mm and their thickness have been measured using a digital micrometer to calculate the cross-sectional area. Two sites of each sample were fixed with two clamps of the tester then the test was started with a stretch speed of 1 mm·min^−1^. Once the sample was completely broken, the stretch stopped automatically. The obtained results are presented as elongation at break, ultimate tensile strength and Young’s Modulus by calculating the average results of 5 different measurements for each type of sample.

### 2.7. Biological Evaluation of the CAP Treatment on Ovine Amniotic Epithelial Stem Cells (oAECs)

#### 2.7.1. Ethic Statement

The cells used in this research were obtained from the amniotic membranes of slaughtered Appenninica breed sheep, considered as waste reproductive tissues of animal slaughtered for food purposes. For this reason, no ethical statement is required.

#### 2.7.2. Cell Isolation and Characterization

Fetuses of 25–35 cm of length, at approximately 2–3 months of pregnancy, were used to collect the amniotic membranes and hence isolate cells as previously described [91]. Briefly, the enzymatic digestion technique with 0.25% Trypsin-EDTA (Sigma Chemical, St. Louis, MO, USA) was applied to collect the ovine AECs (oAECs) from the epithelial layer of the amniotic membrane [91]. The enzymatic activity was stopped by adding fetal bovine serum (FBS) at a final concentration of 10% and the cell suspension was filtered through a 40 µm cell filter. After centrifugations, the pellet was resuspended, and cells were counted by using a hemocytometer chamber after being stained with Trypan-Blue dye to determine the number of viable cells. Cells at a concentration of 3 × 10^3^ cells/cm^2^ were cultured on Petri dishes containing culture medium composed by Minimum Essential Medium Eagle-α (α-MEM) supplemented with 20% FBS, 1% ultraglutamine, 1% amphotericin, and 1% penicillin/streptomycin and incubated at 38 °C with 5% CO_2_. Reached 70% of confluence, oAECs were dissociated with 0.05% Trypsin-EDTA and used for the further experiments. Before their use, the oAECs were characterized for their negativity for hemopoietic markers (CD14, CD58, CD31, and CD45), positivity for both surface adhesion molecules (CD29, CD49f, and CD166) and stemness markers (TERT, SOX2, OCT4, and NANOG), their low expression for MHC class I molecules, the absence of MHC class II (HLA-DR) antigens, as described in previous reports [84,88,90,91] and their negativity for tendon related genes Scleraxis (*SCX*), collagen type I (*COL1*), and tenomodulin (*TNMD*) [90].

#### 2.7.3. Scaffold Sterilization and Cell Seeding

Untreated (PLGA) and CAP treated PLGA microfibers were sterilized before their uses for biological investigations. In detail, rectangular pieces of electrospun PLGA microfibers with dimensions 15 mm × 7 mm were sterilized in 70% ethanol (EtOH) prepared in 0.9% NaCl/distilled water (diH_2_O) for 10 s, hydrated then with sterile phosphate buffer saline (PBS) as described previously [64]. Further, the fleeces were incubated in cell culture growth medium (GM) for 24 h at 38 °C with 5% CO_2_.

To assess the effect of CAP treatment on the biological response of oAECs, cells were seeded onto the different PLGA microfibers at a density of 0.05 × 10^6^ cells per sample. The cells were kept in the incubator for 2 h before adding 1 mL/each sample of GM composed of α-MEM supplemented with 10% FBS, 1% ultraglutamine, 1% amphotericin, and 1% penicillin/streptomycin and incubated at 38 °C with 5% CO_2_. After seeding, the PLGA and CAP treated PLGA microfibers placed onto glass discs were incubated in GM at 38 °C with 5% CO_2_ for 24 and 48 h. For all experiments, oAECs cultured on Petri dishes were used as control.

#### 2.7.4. Effect of CAP Treatment on oAECs Viability

To assess cell viability, a live/dead cell staining was performed after 24 and 48 h of culture. Cells on Petri dishes and onto untreated and CAP treated microfibers (*n* = 3 for each fleece type/ time point) were incubated with Calcein AM, a viable cell green fluorescent dye, at a concentration of 4 µM for 30 min, followed by Hoechst 3342 at a final concentration of 1:2000 for 15 min to counterstain cell nuclei. After 20 min, samples were incubated with Propidium Iodide, a dead cell red fluorescent dye, at a concentration of 12 µM and mounted directly for fluorescence microscope observation. The images were captured using a Nikon Ar1 laser confocal scanning microscope (Nikon, Düsseldorf, Germany) equipped with the NIS-Element software, using a Plan Apo λ 40× oil objective (numerical aperture 1.3; zoom 1.00×; Refractive Index: 1.515; pinhole size: 12.8 µm; pixel size: 0.63 µm; 1 picture every 0.15 s). The used channels are as follows:

Channel 1: DAPI; λ_exc_ = 404 nm; λ_em_ = 450/50 nm, at 81% of the maximum laser power.
Channel 2: FITC; λ_exc_ = 488 nm; λ_em_ = 525/50 nm, at 3.1% of the maximum laser power

Cell viability was determined by counting Calcein-AM positive cells/100 total cells and propidium iodide positive cells/100 total cells counterstained with Hoechst 3342.

#### 2.7.5. Effect of CAP Treatment on oAECs Proliferation

To assess the effect of CAP treatment on oAECs proliferation, immunocytochemical analysis (ICC) was performed to evaluate the expression of Ki-67, a nuclear marker associated with cell proliferation. Cells seeded onto Petri dishes, on the untreated and CAP treated PLGA microfibers were quantified for their Ki-67 positivity after 24 and 48 h of culture (*n* = 3 for each fleece type/ time point). In detail, at these time points, cells were fixed with 4% paraformaldehyde for 15 min and washed thrice with PBS before being permeabilized with PBS/ 0.1% Triton X-100 for 5 min. After washing in PBS, the non-specific binding sites were blocked with PBS/1% BSA for 1 h. Afterwards, cells were incubated overnight at 4 °C with anti-Ki-67 primary antibody (Dako Cytomation, Glostrup, Denmark) diluted 1:50 in PBS/1% BSA. Primary antibody was revealed by using an anti-mouse Alexa Fluor 488 secondary antibody (Molecular Probes) diluted 1:100 in PBS and incubated for 1 h. Cell nuclei were counterstained with DAPI diluted 1:5000 in PBS (Vectastain, Burlingame, CA, USA). The primary antibody was replaced non-immune sera as negative control. All controls were negative.

The quantification of the proliferation index (PI) was determined by counting Ki-67 positive cells/100 cells counterstained with DAPI.

For image acquisition, an Axioskop 2 Plus incident-light fluorescence microscope (Carl Zeiss) equipped with a CCD camera (Axiovision Cam; Carl Zeiss) possessing a resolution of 1300 × 1030 pixels, and interfaced to a computer workstation provided with an interactive and automatic image analyzer (Axiovision, Carl Zeiss) was used. Digital images were captured at ×100 and ×200 magnification using standard filters set up optimized for FITC or DAPI.

#### 2.7.6. Effect of CAP Treatment on oAECs Penetration and Cell Count within PLGA Microfibers

Cells seeded for 24 and 48 h of culture onto Petri dishes, untreated and CAP treated PLGA microfibers (*n* = 3 for each fleece type/ time point) were incubated with Phalloidin (Sigma-Aldrich, St. Louis, MO, USA) for F-actin filament stain to assess cells’ distribution and penetration within the electrospun materials. In brief, after fixation in 4% paraformaldehyde for 15 min, cells were permeabilized with PBS/0.1% Triton X-100 for 10 min at room temperature (RT). After being washed with PBS, phalloidin-TRITC (dilution 1:10 in PBS; Sigma Aldrich, Missouri, USA) was incubated with each sample for 20 min followed by a nuclear counterstaining with DAPI (dilution 1:5000 in PBS; Vectastain) for 15 min at RT. The images were captured using a Nikon Ar1 laser confocal scanning microscope (Nikon, Düsseldorf, Germany) as described in the section above with the followed channels:

Channel 1: DAPI; λ_exc_ = 404 nm; λ_em_ = 450/50 nm, at 81% of the maximum laser power.
Channel 2: TRITC; λ_exc_ = 561.5 nm; λ_em_ = 595/50 nm, at 0.6% of the maximum laser power.

Cell penetration for oAECs cultured onto untreated and treated electrospun PLGA microfibers was investigated using the XZ projection acquired from confocal microscopy Z-stacks at a magnification of ×400, on which it was applied the depth-coded MaxIP (Maximum Intensity Projection). This analysis option allows the generation of a color gradient to the pixels on the images with the highest intensity values of the Z-sequences to determine cell penetration property within PLGA microfibers [10,25]. Cell penetration was quantified on depth coded MaxIP modified images by counting cell nuclei counterstained with DAPI of untreated and CAP treated electrospun PLGA microfibers (3 different fields/3 different samples/each group). In detail, the Z-stack acquisitions were divided into different layers (each layer had a thickness of 10 µm) on which the cell quantification was carried out. The results were expressed in percentage respect to the total cell count within the different Z-stack layers. Cellularity was calculated as the total number of DAPI stained nuclei inside a standard field of ×400 magnification from five images of three different samples for each sample type [90].

#### 2.7.7. Teno-Inductive Properties of the CAP Treated PLGA Microfibers

To investigate if the CAP treatment would maintain the teno-inductive properties of the fabricated electrospun PLGA microfibers; cells seeded onto untreated (PLGA) and CAP treated PLGA microfibers were assessed for their expression of tenomodulin (TNMD), a mature tendon marker. After culture for 24 and 48 h, cells (*n* = 3 for each fleece type/ time point) were fixed in paraformaldehyde for 15 min then washed with PBS before being permeabilized with PBS/0.1% Triton X-100 for 10 min. The non-specific bindings were blocked with PBS/1% BSA for 1 h and anti-TNMD antibody (dilution 1:50 in PBS/1% BSA; Biorbyt, Cambridge, UK) was added and incubated overnight at 4 °C. The day after, the secondary antibody anti-rabbit Alexa Fluor 488 (dilution 1:400 in PBS/1% BSA; Molecular Probes, Göteborg, Sweden) was incubated for 1 h to reveal the primary antibody. Cell nuclei were counterstained with DAPI (dilution 1:5000 in PBS, Vectastain) and the samples were imaged under an Axioskop 2 Plus incident-light fluorescence microscope (Carl Zeiss, Oberkochen, Germany) as described above. Negative control haw been used by replacing the primary antibody with non-immune sera. All controls were negative.

### 2.8. Statistical Analysis

Considering the physicochemical analyses, three replicates were considered for each analysis type except for the mechanical tests where five samples were analyzed for each sample group. For the biological investigations, the analyses have been carried out on oAECs of at least three fetuses (*n* = three biological replicates) by analyzing quantitative data of each sample in triplicate for each analysis. The results were represented as average ± standard deviation (S.D.). D’Agostino and Pearson tests were used to assess the normal distribution of the results. Data set comparison was performed by One-way ANOVA multi-comparison tests followed by Tukey post hoc tests using GraphPad 8 (GraphPad Software, San Diego, CA, USA). Value at least *p* < 0.05 was considered significant.

## 3. Results and Discussion

### 3.1. Morphological Analysis of the Electrospun PLGA Microfibers

**Figure 2:**
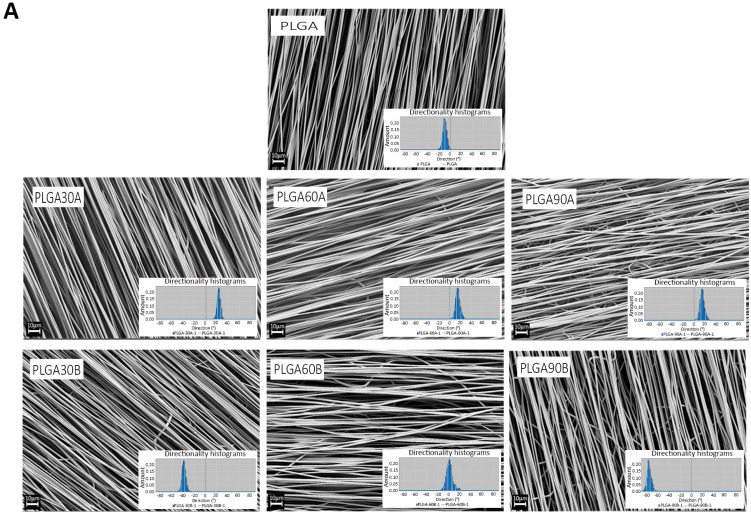

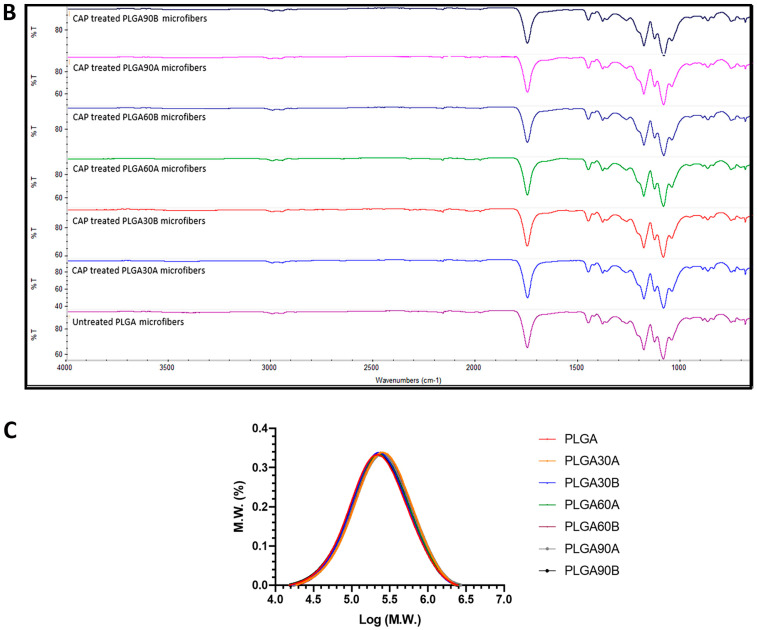
(**A**) SEM investigations of the untreated and plasma-treated electrospun microfibers and the distribution of the fiber orientation. The SEM images showed that the CAP treatment did not affect the morphology neither the alignment of the electrospun microfibers (*p* > 0.05). The insets represent the histograms of each sample showing the angle distribution of the electrospun fibers on untreated and CAP treated PLGA samples. It can be noticed that fiber alignment after CAP treatment, by varying the plasma process’ parameters (exposure time and working distance), was maintained as the curves obtained from the analyses showed a sharp Gaussian curve confirming the aligned topography of the microfibers. Scale bars = 10 mm. (**B**) Infrared spectra for untreated and CAP treated PLGA microfibers at different exposure times (30, 60, and 90 s) and working distances (1.3 and 1.7 cm) reported in the 650 and 4000 cm^−1^. (**C**) Molar mass distribution of untreated and CAP treated electrospun PLGA microfibers.

The CAP treated electrospun PLGA microfibers did not show macroscopically any sign of cratering or defect. Microscopically, the SEM micrographs of the untreated and plasma treated electrospun PLGA microfibers are shown in Figure 2A. The obtained PLGA scaffolds show bead- and defects-free aligned fibers. The plasma treatment did not alter the surface topography of the scaffolds even after long exposure treatment time (90 s) from the smaller gap between the scaffold’s surface and the plasma pen (1.3 cm). Table 2 shows the average fiber diameter sizes of the electrospun PLGA microfibers ranging between 1.335 ± 0.015 and 1.398 ± 0.011 µm (*p* > 0.05) confirming that CAP treatment does not affect the treated PLGA microfibers since no significant changes were observed compared to the untreated ones (*p* > 0.05).

Concerning fiber alignment, no significant changes have been noticed after N_2_ CAP treatment compared to the untreated one. In detail, the Fourier power spectra analyses conducted on the samples exhibited sharp Gaussian curve shapes (Figure 2A) with a goodness of fit values ranging between 0.94 and 1 and dispersion values, which report the standard deviation of the Gaussian, from 13.55 ± 0.14° to 4.30 ± 0.17° (Table 2).

It can be concluded, that plasma treatment did not alter fibers organization, their point-bonded junctions and no melting effect was observed that in turn may cause physical damage to the electrospun PLGA microfibers. These results are in accordance with previous study where electrospun PLLA scaffolds have shown no alteration in their ultrastructure after 3 min of plasma treatment with nitrogen [56]. However, these results may reflect also the effect of the type of plasma reactive gas on the treated materials since Park et al., [59] found differences in the morphology of PLGA scaffolds after plasma treatment with oxygen and ammonia. In detail, they found that PLGA microfibers exposed to oxygen plasma for 180 s lost their structure while no significant changes on the PLGA microfibers were seen when ammonia plasma treatment was applied [59].

### 3.2. Chemical Characterization of the Electrospun PLGA Microfibers

The effect of the plasma treatment on the surfaces on PLGA microfibers and chemical functional groups has been investigated. FTIR spectroscopy has been performed on the untreated and CAP treated electrospun PLGA microfibers and the obtained spectra are shown in Figure 2B. Absorption bands for the ester carbonyl group (C=O) and ether group (C-O-C) at approximately 1748 and 1085 cm^−1^ were present in the FTIR spectra characterizing the untreated electrospun PLGA microfibers. The peaks appearing at 1452 and 1044 cm^−1^ could be attributed to asymmetric stretching vibration and symmetric stretching vibration of C-H and C-CH_3_, respectively. Concerning the CAP treated PLGA microfibers, no FTIR peak shift or new peaks have been found compared to the neat electrospun PLGA microfibers.

The fact that no changes were observed in the FTIR spectra of the treated materials could be related to the changes that only occur superficially in order of nanometers during plasma treatment despite the analysis of FTIR technique is from 1 to 5 µm in depth [100]. These results are in accordance with other reported data where no changes have been detected in the FTIR spectra after plasma treatment [47,56,62].

### 3.3. Molecular Weight Determination

The effect of CAP plasma on the PLGA microfibers was monitored in terms of molar mass by means of gel permeation chromatography (GPC). Figure 2C shows the molar mass distribution of the PLGA microfibers, represented as Log (M.W.), before and after nitrogen CAP treatment for different exposure treatment time and from different working distances. The untreated PLGA microfibers exhibited a unique modal Gaussian-like distribution showing a sharp peak at 5.4 g·mol^−1^ that corresponds to M.W. = 258,000 g·mol^−1^. After CAP treatment, no displacement or shift of the curves were noticed and they were similar to that obtained for the untreated PLGA materials confirming that the treated PLGA materials were not degraded after CAP treatment and the long exposure time (90 s) as well as the short working distance (1.3 cm) did not affect negatively the integrity of the copolymer neither its properties.

### 3.4. XPS Analysis

**Figure 3:**
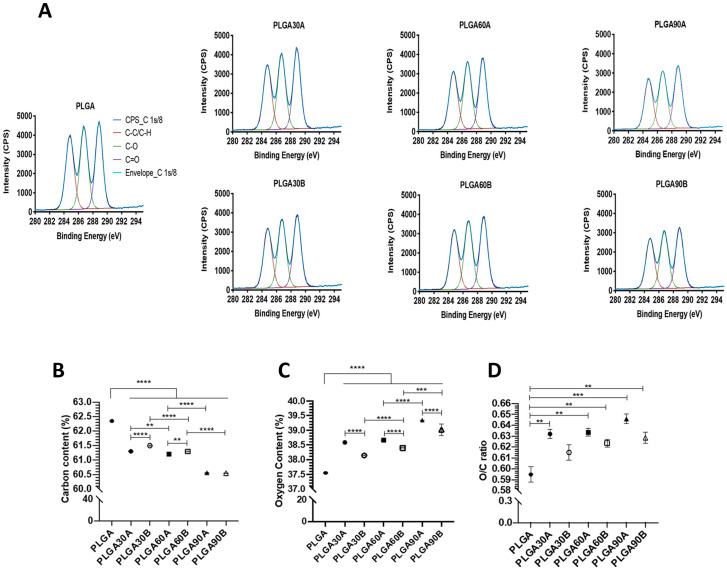

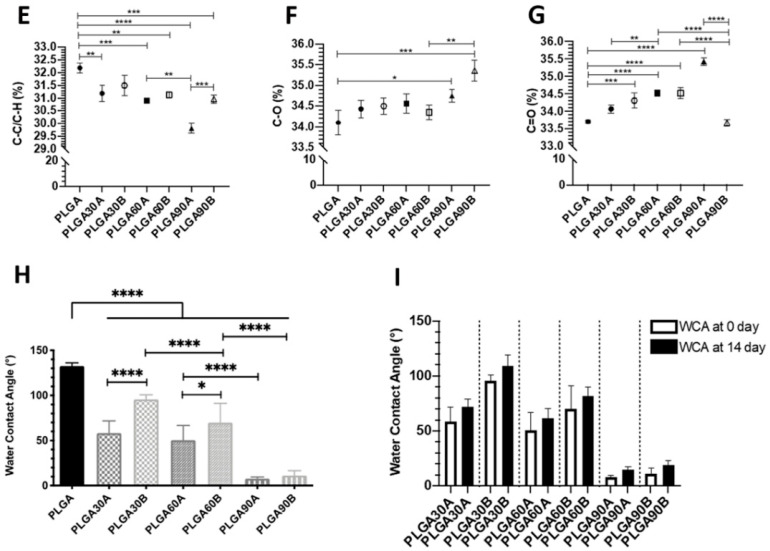
(**A**) The deconvoluted C1s XPS spectra of the untreated and CAP treated electrospun PLGA microfibers with different process’ parameters. Atomic content of (**B**) carbon, (**C**) oxygen and (**D**) O/C ratio obtained by XPS technique for the untreated and CAP treated PLGA microfibers. Bonds percentages of the C1s deconvolution regarding (**E**) C-C/C-H, (**F**) C-O and (**G**) C=O obtained by XPS technique for the untreated and CAP treated PLGA microfibers. (**H**) Changes in the Water contact angles (WCA) between untreated and CAP treated PLGA microfibers by varying the exposure time and working distance. (**I**) Ageing analysis to assess the stability of the CAP treated PLGA microfibers between day 0 and day 14. *, **, ***, and **** Statistically significant between the groups (*p* < 0.05, *p* < 0.01, *p* < 0.001, and *p* < 0.0001, respectively).

The elemental composition of the electrospun PLGA microfibers before and after CAP treatment has been assessed using the XPS technique. The surface activation of the electrospun PLGA microfibers after plasma treatment is attained by the incorporation of oxygen containing species as observed in Figure 3A. In detail, three carbon types can be detected from the deconvolution of the C1s signal of untreated and CAP treated PLGA microfibers that can be assigned to the C-C/C-H bonds of the hydrocarbon chain at 284.8 eV, the C-O and C=O bonds at 286.77 and 288.86 eV, respectively.

In detail, in Figure 3B,C, the carbon and oxygen content in the untreated electrospun PLGA microfibers were 62.35% and 37.56%, respectively. Treated via CAP, the carbon content decreases gradually and significantly until reaching its lowest value (~56%) after 90 s from both working distances (*p* < 0.0001). Moreover, the working distance has shown a significant effect on decreasing the carbon content only after 30 and 60 s from the working distance of 1.3 cm compared to 1.7 cm (*p* < 0.0001 for 30 s and *p* < 0.01 for 60 s). The increase in the exposure treatment time led to a further significant decrease in the carbon content (*p* < 0.01).

In turn, by increasing the treatment exposure time, the gradual decrease in carbon content was accompanied with a significant increase in the oxygen content that reaches a maximum of 39.34% after 90 s compared to the untreated PLGA microfibers (37.56%, *p* < 0.0001, Figure 3B,C). Moreover, the working distance of 1.3 cm seemed to have better effect on increasing oxygen content for all treatment exposure time compared to 1.7 cm (*p* < 0.0001), since the free radicals generated from the activated plasma interact faster in the air atmosphere with the radicals formed on the surface of the PLGA microfibers.

The value of O/C ratio increased in the CAP treated PLGA microfibers by increasing the treatment exposure time with the highest value of around 0.65 obtained after 90 s from a working distance of 1.3 cm compared to the untreated ones (0.59, *p* < 0.01). Similarly, the O/C ratio represents a trend confirming the effect not only to the exposure time but also to the working distance on increasing the hydrophilic properties of the PLGA microfibers (Figure 3D). Performing the CAP treatment from a lower working distance 1.3 cm rather than 1.7 cm favored the generation of functional oxygen groups represented by the increase of oxygen content and the O/C ratio (Figure 3C,D).

More specifically, the content of C-C/C-H bonds tended to decrease for at least 13% after plasma treatment (Figure 3E, *p* < 0.01). As shown in Figure 3E, the C-C/C-H content decreased gradually by increasing CAP exposure time. The lowest C-C/C-H percentage (29.35%) has been obtained after 90 s from a working distance of 1.3 cm compared to 30.56% from the 1.7 cm working distance treated for the same exposure time (*p* < 0.001 Figure 3E). In Figure 3F,G, the content of C-O bonds increased gradually after CAP treatment by increasing treatment exposure time whereas significant higher percentages compared to the untreated microfibers were found only after 90 s of treatment from either working distances (*p <* 0.01). The increase in single carbon bond to oxygen (C-O) content in the CAP treated PLGA microfibers compared to the untreated ones could be attributed to the formation of hydroxyl or peroxyl groups on the surface of PLGA microfibers in accordance with the results obtained previously [59]. Concerning the content of C=O bonds, there was a proportional increase with the treatment time. The higher is the exposure time, the higher is the C=O content, except for the treatment done for 90 s from the working distance of 1.7 that showed the lowest C=O percentage compared to other treated groups (*p* < 0.001). Interestingly, the high C-O and C=O percentages were observed after 90 s from working distance of 1.7 and 1.3 cm, respectively.

Altogether, from the obtained results of the XPS investigations (Figure 3A–G), it can be noticed that the significant increase in oxygen containing functionalities on the plasma-treated surfaces of PLGA microfibers is considered the main parameter that improved the hydrophilic properties of the treated surfaces [101] and may be the reason for improving cell adhesion. Delivering sufficient oxygen to the transplanted cells is one of the most critical issues that affects cell survival and biology of engineered tissues. In this research, it was found that the treatment with the lower working distance (1.3 cm) and for longer time (90 s) favored an increase of oxygen content and a decrease of carbon content as evident also by the O/C ratio. Moreover, the C-O and C=O, mainly formed by plasma-initiated reactions with the C-C and C-H bindings, which resulted in a linear oxygen increase content that was dependent on the time of exposure and by the lower working distance. The increase of oxygen content within the treated samples despite the absence of nitrogen functional groups such as amine and amide could be attributed to the fact that the CAP treatment was not done under vacuum conditions and the samples were subjected before, during and after the plasma treatment to the atmospheric air. In this study, at the outlet of the plasma nozzle, the nitrogen plasma jet was then mixed with air. When the CAP treated electrospun PLGA microfibers are subjected to the air, the formed radical will mainly react with oxygen. It can be hypothesized that the accelerated and bombarded ions generated from lower working distance led to the formation of highly reactive radicals with long lifetime on the surface of the treated samples that in turn reacted with the oxygen presented in the air favoring its interaction with the treated materials rather than nitrogen [102]. These results are in accordance with a previous work where no N-containing functional groups were observed on the surface of PLGA films after being treated with N_2_ atmospheric plasma and the enhanced hydrophilic properties were improved by the slight increase of oxygen content compared to the non-treated materials [103]. Moreover, Sanchis et al. confirmed that also under vacuum conditions, no additional nitrogen functionalities can be detected on the surface of polyurethane scaffolds after N_2_ plasma treatment [98].

However, as reported by Gholipourmalekabadi et al. [101], the main concern associated with high oxygen content in a construct could be the production of toxic agents such as hydrogen peroxide, residual reactive oxygen species, and salts as decomposition byproducts.

### 3.5. Effect of CAP Treatment on the Evaluation of WCA of the Electrospun PLGA Microfibers

In order to confirm the improvement of the hydrophilic properties of the CAP treated PLGA microfibers compared to the untreated ones, the contact angles between the water droplets and the microfibers surfaces have been measured. As shown in Figure 3H, it can be seen a strong dependence between the water contact angle of the samples and the process parameters used. Both exposure time and working distance affected significantly the hydrophilic properties of the PLGA microfibers. In detail, the untreated electrospun PLGA microfibers exhibited high water contact angle (132 ± 3.67°) confirming their high hydrophobic property and the difficulty to absorb water. On the contrary, previous experiments conducted on PLGA films revealed a less hydrophobic state with a WCA of around 80° [44,45,69,104] compared with the untreated electrospun PLGA microfibers produced in this study.

After CAP treatment, the water contact angle dropped significantly to reach the lowest values of 7.81 ± 1.86° with the CAP treatment done from a distance of 1.3 cm for 90 s (*p* < 0.0001). In addition, the variation in the working distance (1.3 vs. 1.7 cm) showed significantly different results between the groups treated for 30 and 60 s, respectively (*p* < 0.0001 and *p* < 0.05, respectively). After 30 and 60 s of CAP treatment, the PLGA microfibers treated from 1.3 cm showed significantly lower WCA values of around 39% and 28%, respectively, compared to those treated from 1.7 cm. At 90 s treatment, the distance seemed to have no effect on the water contact angle (*p* > 0.05). Moreover, when treated from a distance of 1.7 cm, WCA within the CAP treated PLGA microfibers decreased significantly by increasing the treatment exposure time from 30 to 90 s (*p* < 0.0001), whereas when the PLGA microfibers were treated from a distance of 1.3 cm, the significant decrease in WCA was noticed only when the exposure time increased from 60 to 90 s (*p* < 0.0001).

These results confirmed also the hydrophilic properties of CAP treated microfibers through the WCA measurements since the changes in process parameters strongly influence the solid-liquid interface. The high WCA obtained with the untreated PLGA microfibers revealed its high hydrophobic properties compared to the treated ones. When increasing the treatment exposure time and decreasing the treatment working distance, the WCAs of the PLGA microfibers decreased gradually from around 133° to reach an average of 8° after 90 s of treatments. The decrease in WCA after N_2_ plasma treatment may be explained by the exothermic reaction that occurred between the plasma generated ions and the PLGA electrons, which interact to form free nitrogen radicals. The released energy produced by the exothermic reaction seems to be sufficient to break the C-C and C-H bonds allowing the formation of new hydrophilic bonds on the surface of the CAP treated PLGA microfibers. In fact, it was found that the treatment with the lower working distance (1.3 cm) and for longer time (90 s) favored an increase of oxygen content and a decrease of carbon content as evident also by the O/C ratio. As a confirmation, PLGA microfibers treated for 90 s with 1.3 cm had the lowest WCA values, as also reported with a previous work [103]. However, of note, a high rate of hydration can remarkably affect the quality and quantity of oxygenation [101].

Bolbasov et al. treated electrospun PLLA scaffolds with plasma using nitrogen as working gas for different treatment time (1, 2, 4, 6, and 8 min) and they found that the WCA decreased after 1 min from 129° to 20° and re-increase with the increase of treatment time from 2 to 8 min to reach 50° [56]. An improved hydrophilicity was observed on the PLGA films (~90 ± 2.3°) compared to PLGA fibers (133 ± 3.3°) in a comparative study conducted by Wang et al. [104]. This variation in WCA between the unique structurally PLGA material and the high hydrophobic profile observed on electrospun fibers compared to films could be explained by the reduced pore size and increased fiber inter-junctions in electrospun microfibers that greatly hinder the air penetration and thus result as an obstacle for water infiltration [93].

Therefore, increasing the surface wettability of electrospun PLGA microfibers using N_2_ plasma may influence positively the cells cultivated on the treated electrospun microfibers. However, the optimum designed scaffold for tendon tissue engineering must maintain a controlled degradation rate allowing in turn the formation of neo-tissue instead.

### 3.6. Ageing Effects of Nitrogen Plasma on Treated PLGA Microfibers

After being activated with plasma, the surfaces of PLGA microfibers can lose partially their hydrophilicity due to the reorientation of the integrated polar groups towards the material bulk as well as due to the reactions that occur after plasma treatment between the modified surfaces and present atmospheric components (CO_2_ and H_2_O) [105,106]. In order to assess the stability and the efficiency of the CAP treatment on the surfaces of the PLGA microfibers, the treated samples with different conditions have been conserved at room temperature and analyzed after 14 days to control the ageing effect and compared to those obtained at day 0 (Figure 3H,I).

The obtained results showed an increase of about 13, 11, and 7% of the WCA for those treated for 30, 60, and 90 s, respectively at 14 days compared to day 0 (*p* > 0.05). Only the electrospun PLGA microfibers treated for 30 s from 1.7 cm working distance lost 35% of their initial WCA after treatment compared to the other treated groups. It seems that the samples treated for longer time maintained better their hydrophilic properties due to the polar functionalities on its surface. These results could be explained by the fact that the chain alignment of the treated aligned fibers might obstruct the movement and re-orientation of the integrated polar groups to the material bulk also supported by the results obtained previously [93]. It could be also hypothesized that the aligned topography of the electrospun microfibers resist to the ageing effect due to the inter-fibers junctions as supported by previous study [93].

In contrast to the obtained results, in a research conducted previously studying the ageing effect of N_2_ plasma treatment on PLLA scaffold, the authors showed a 76% loss in the treatment efficiency after 26 days of treatment [106].

### 3.7. CAP Treatment affects the Mechanical Properties of the PLGA Microfibers

**Figure 4:**
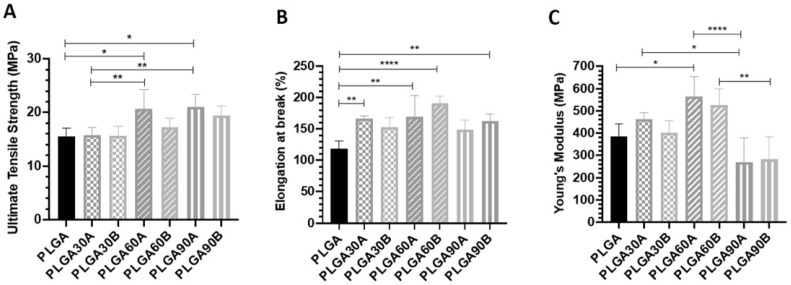
Mechanical properties (**A**) Ultimate Tensile Strength, (**B**) Elongation at break, and (**C**) Young’s Modulus of the untreated and CAP treated PLGA microfibers. *, ** and *** Statistically significant between the groups (*p* < 0.05, *p* < 0.01, and *p* < 0.001, respectively).

To assess the effect of CAP treatment as well as the changing in process parameters on the mechanical properties of the electrospun PLGA microfibers, the ultimate tensile strength (UTS), elongation at break and Young’s modulus were determined and the mean values are shown in Figure 4. The untreated PLGA microfibers exhibited lower UTS and elongation at break values compared to the treated ones. No significant changes in the UTS have been observed after 30 s treatment from either treatment working distances (*p* > 0.05). In contrast, by increasing the exposure time to 60 and 90 s, the UTS increased gradually and significantly compared to the untreated PLGA microfibers treated from a working distance of 1.3 cm (20.7 ± 3.6 and 21.1 ± 2.3 MPa vs. 15.5 ± 1.7 MPa for PLGA60A and PLGA90A vs. PLGA, respectively). The changing in the working distance did not alter significantly the UTS properties of the PLGA microfibers exposed to plasma for the same treatment time (*p* > 0.05). Considering the short working distance between the plasma source and the material surface, 30 s of CAP treatment seemed to be not sufficient for increasing significantly the UTS of the PLGA microfibers as after 60 and 90 s of exposure (PLGA60A: 20.7 ± 3.6 and PLGA90A: 21.1 ± 2.3 MPa vs. 15.7 ± 1.4, *p* < 0.01).

The elongation at break augmented gradually and significantly by increasing CAP treatment exposure time to reach a maximum after 60 s and decrease again after 90 s of treatment (*p* < 0.01). In detail, after 30 s of treatment, the elongation at break increased from both distances (1.3 and 1.7 cm) and only significantly from the shorter distance compared to the untreated materials (PLGA30A: 166.6 ± 4.756% vs. 118.2 ± 12.8%, *p* < 0.01). By increasing the exposure treatment time to 60 s from either both working distances, the elongation at break increased significantly to reach 169.7 ± 33.4% for PLGA60A (*p* < 0.01) and 191 ± 10.87% for PLGA60B (*p* < 0.0001) compared to untreated PLGA. Interestingly, after 90 s of treatment, the elongation at break property decreased again while maintaining values higher than that obtained with the untreated PLGA microfibers with a significant higher value treated with an exposure distance of 1.7 cm (*p* < 0.01).

The Young’s modulus property followed a similar trend as for the elongation break since it increased gradually to reach a maximum after 60 s of treatment then decrease to values lower than that obtained with the untreated PLGA microfibers after 90 s. An increase in the Young’s modulus has been observed after 30 s of CAP treatment with higher values obtained from the shorter working distance 1.3 cm (*p* > 0.05). These values tend to increase after 60 s to reach their maximums with a significant increase compared to the neat PLGA microfibers when microfibers were CAP treated from 1.3 cm working distance (*p* < 0.05). Remarkably, after being exposed for 90 s from both working distances, PLGA microfibers lost significantly their Young’s modulus property compared to those treated for 60 s (PLGA60A: 564 ± 91.34 MPa vs. PLGA90A: 270.2 ± 109 MPa, *p* < 0.0001 and PLGA90A: 525 ± 72.24 MPa vs. 284.6 ± 100.4 MPa, *p* < 0.01). The Young’s modulus values obtained after 90 s of treatments from either working distances showed lower values than those of the untreated materials (*p* > 0.05).

It seems that the exposure time is the main factor affecting the mechanical properties of the treated PLGA microfibers rather than the working distance. At higher exposure time (90 s), the fibers might interact together through such reaction of crosslinking that in turn can alter the inter-fiber junctions and hence results in the decrease of the Young’s modulus and elongation at break properties. It could be noticed a bell-like trend for these two parameters that increase to an optimum value after 60 s of CAP treatment then decrease to reach similar or lower values compared to the untreated materials. In contrast, Bolbasov et al. showed that N_2_ plasma treatment on the electrospun PLLA scaffolds did not affect their mechanical properties even after long treatment time (8 min) [56]. The same results were obtained by Wang et al. who observed that increasing air plasma exposure time (60, 120, and 180 s) did not affect the mechanical properties of the electrospun PLGA scaffolds [60].

### 3.8. CAP Treatment of Electrospun PLGA Microfibers Increases Cell adhesion and Penetration Maintaining their Biocompatibility and Tenoinductive Properties on oAECs

**Figure 5:**
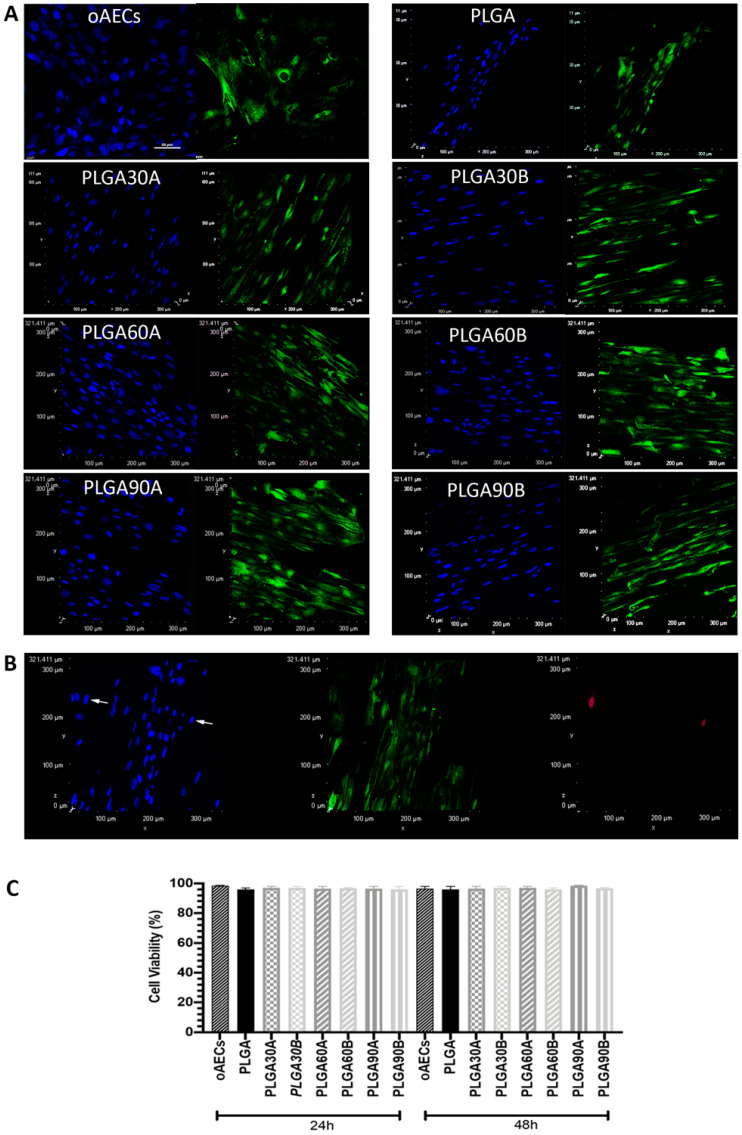
Ovine amniotic epithelial stem cell (oAECs) viability on Petri dishes, untreated and CAP treated electrospun PLGA microfibers. (**A**) Representative images of oAECs survival on Petri dishes, untreated PLGA and CAP treated PLGA microfibers stained with Calcein AM/propidium iodide stains (green and red fluorescence, respectively) after 48 h of culture. Nuclei were counterstained with Hoechst 3342 (blue fluorescence). In these figures the red channel (TRITC) is not shown since the stained cells were all negative to propidium iodide (dead cell marker, red fluorescence) confirming the high vitality of oAECs within the different groups of PLGA microfibers; scale bar = 50 µm. (**B**) Representative image showing few red dead nuclei (propidium iodide, red fluorescence) in electrospun PLGA microfibers. (**C**) Histogram showing oAECs viability of cells cultured on Petri dish (oAECs), seeded on untreated PLGA (PLGA) and different CAP treated PLGA microfibers, after 24 h and 48 h days of culture. All untreated and CAP treated PLGA microfibers are biocompatible for oAECs since no statistical difference was evident among the different groups (*p* > 0.05); (*n* = 3 for each type of fleece/time point, fleece size: 15 mm × 7 mm).

The biocompatibility of the CAP treated PLGA microfibers was assessed on oAECs and compared to untreated (PLGA) microfibers, whereas oAECs seeded on Petri dishes were used as internal control, using the alive and dead cell markers, Calcein AM and propidium iodide, respectively. After 24 h and 48 h of culture, the cells were alive on all PLGA microfibers types (*p* > 0.05, Figure 5A,C) and only few cells, about 1%, were positive to propidium iodide (Figure 5B), showing that CAP did not alter PLGA biocompatibility for oAECs.

**Figure 6:**
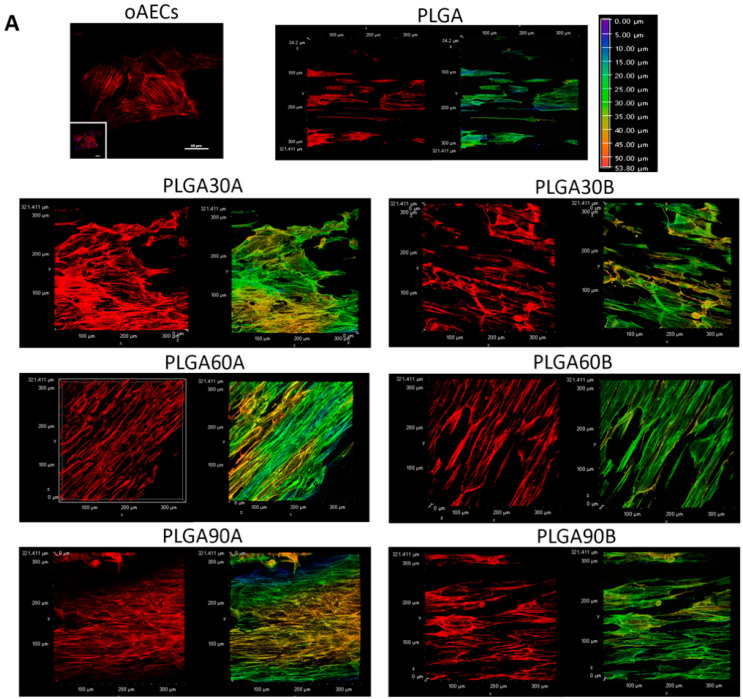

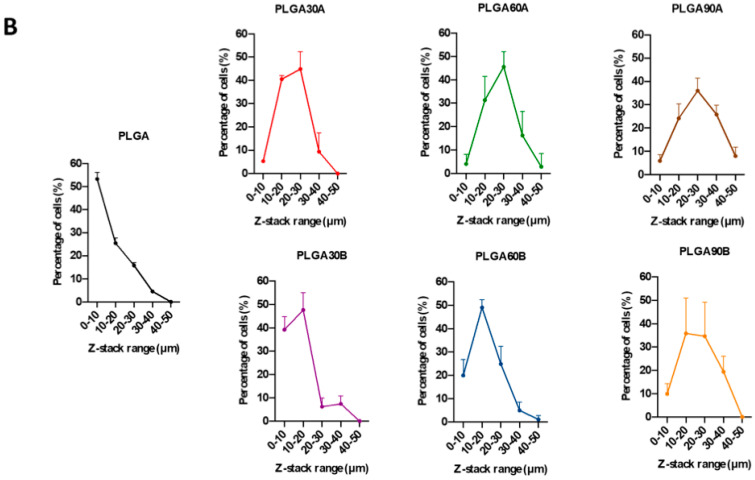
Ovine amniotic epithelial stem cell (oAECs) distribution on untreated and treated PLGA microfibers. (**A**) On the left, representative images assessing oAECs positivity to phalloidin stain (red fluorescence) in oAECs cultured on Petri dishes (scale bar = 50 µm) or on untreated (PLGA) and different CAP treated PLGA (PLGA30A, PLGA30B, PLGA60A, PLGA60B, PLGA90A and PLGA90B) microfibers after 48 h of culture. On the right, phalloidin marked cells were analyzed with the MaxIP. Representative XY confocal images showing oAECs distribution within untreated (PLGA) and different type of CAP treated PLGA microfibers. The depth coded MaxIP analysis was used to assess cell penetration within the microfibers by defining the gradient color related to the direction of the cytoplasm of the cells. The purple color of the gradient scale refers to the surface of the microfibers, while the red color is attributed to the bottom of the surface. Thus, the cytoplasm of the most superficial cells is shown with a green color, whereas the cytoplasm of the cells localized at the bottom of the microfibers are shown in orange/red. It is evident that oAECs have an optimal distribution within the microfibers, especially in PLGA30A, PLGA30B, PLGA60A and PLGA90A. (**B**) Quantification of cell penetration. Cell penetration was quantified on depth coded MaxIP modified images. The Z-stack acquisitions were divided into 5 layers (10 µm each) (3 different fields/3 different samples/each group).

Moreover, cells were stained with phalloidin (red fluorescence), an actin stain, to verify their penetration within the PLGA microfibers. On the Z-stacks of phalloidin acquisitions, it was carried out the depth coded MaxIP analysis. This analysis automatically defines the gradient color (in purple the surface, whereas in red the bottom) related to the direction of the cytoplasm of cells within the PLGA microfibers (Figure 6A). It was possible to demonstrate an optimal oAECs penetration within the PLGA microfibers especially those. treated from a working distance of 1.3 cm. Although, after 48 h of culture, in PLGA30B and PLGA60B the cells penetrated less (all cytoplasm are shown in green with the depth coded MaxIP analysis; Figure 6A) compared to the other CAP treated PLGA microfibers in which the cells nearly reached the bottom (the cytoplasm of several cells are shown in orange and few in red with the depth coded MaxIP analysis; Figure 6A).

In particular, it was evident from Figure 6B that the most seeded oAECs (about 53%) on untreated PLGA microfibers were distributed superficially (0–10 µm layer), whereas only 4% of oAECs were found in the deep layer corresponding to 30–40 µm. Increasing the hydrophilicity of the PLGA microfibers facilitated oAECs’ penetration. In fact, the CAP treatment effectuated from lower distance (1.3 cm) favored cell penetration compared to the higher distance (1.7 cm) (Figure 6B). More specifically, CAP treatment from a working distance of 1.3 cm showed almost around 40% of cells in the layer of 20–30 µm thickness. Moreover, oAECs were able to reach the deepest PLGA microfibers layer (40–50 µm) after being CAP treated for 60 and 90 s with cell penetration percentages of around 3 and 8%, respectively.

When the electrospun PLGA microfibers were CAP treated from a working distance of 1.7 cm, the majority of cells penetrated within the layer of about 10–20 µm (Figure 6B). By increasing the exposure time, cells were able to penetrate more since about 20% of oAECs reached the layer of 30–40 µm in the case of PLGA90B (Figure 6B).

**Figure 7:**
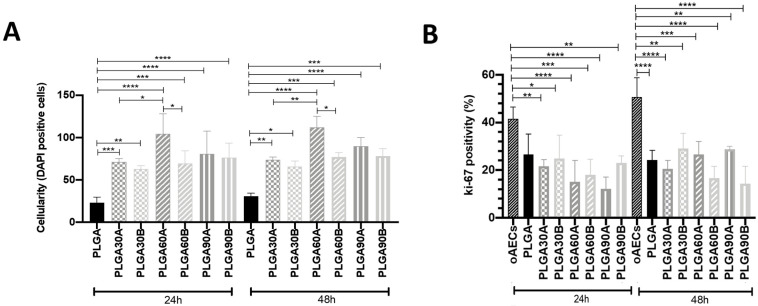

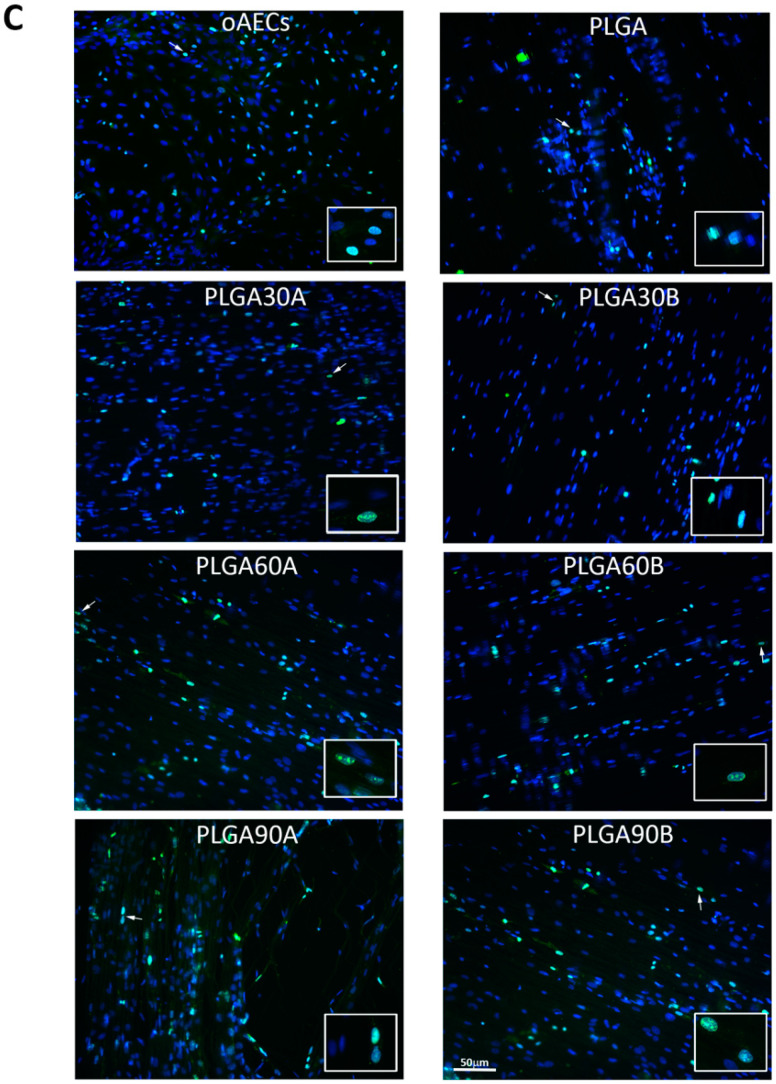
Cellularity and Proliferation index (PI) of untreated and treated PLGA microfibers. (**A**) Cellularity quantified within the analyzed PLGA microfibers, expressed as the total number of nuclei (DAPI stained) in a field of × 400 magnification after 24 h and 48 h of culture. (**B**) Proliferation index (PI) of oAECs cultured on Petri dishes (oAECs) or within the analyzed untreated (PLGA) or treated CAP samples. (**C**) Representative images of oAECs within the analyzed PLGA microfibers and Petri dishes showing Ki-67 positivity (green fluorescence) and cell nuclei counterstained in blue (DAPI); Scale bars = 50 μm. The insets show details of each sample (white arrows indicate the corresponding cells present in the insets at lower magnification) in which it is evident the co-localization of Ki-67 expression and nuclei (*n* = 3 for each type of sample/ time point, sample size: 15 mm × 7 mm). *, **, *** and **** Statistically different values between different studied groups for *p* < 0.05, *p* < 0.01, *p* < 0.001 and *p* < 0.0001, respectively.

Cellularity was then calculated within all samples of PLGA microfibers. It was evident after 24 h of culture a significantly higher cell number onto CAP treated PLGA microfibers respect to the untreated one (*p* < 0.05; Figure 7A). This higher number of cells on different CAP PLGA microfibers was maintained also after 48 h of culture (*p* < 0.05; Figure 7A) especially for the PLGA60A samples. The obtained results demonstrate that CAP treatment increased the ability of the cells to adhere better on the surface of the treated electrospun PLGA microfibers respect to the untreated ones. In particular, the better results in terms of cell penetration and cellularity were obtained with the lower working distance (1.3 cm). These results could be justified by the better hydrophilicity and consequently the higher oxygen content of the PLGA microfibers CAP treated from a working distance of 1.3 cm. Moreover, PLGA60A had a cell penetration profile and cellularity comparable to PLGA90A. However, the decreased Young’s modulus and elongation at break properties within the PLGA90A microfibers can be considered as a drawback since constructs fabricated for tendon tissue engineering should possess mechanical properties that must mimic tendon structure and biomechanics to sustain its regeneration. Moreover, the high hydrophilicity of PLGA90A, accordingly to literature data [107,108], may lead to increased water uptake and hence result in a faster degradation rate of the electrospun microfibers hindering in turn the complete formation of the neo-tissue.

On the contrary, PLGA30B and PLGA60B showed the lowest cell penetration profile and cellularity probably due to their lower oxygen content and hydrophilicity (high WCA) amongst the other CAP treated groups.

To evaluate oAECs’ PI within PLGA samples, a cell proliferation marker, Ki-67, was assessed and quantified. The immunocytochemical analysis on oAECs, engineered on untreated and treated PLGA microfibers or cultured in Petri dishes, as internal control, showed a specific positivity for Ki-67 (green fluorescence) in some cell nuclei (Figure 7C) confirming their mitotic activity. Although, cell proliferation on all samples of PLGA microfibers was not significantly different (*p* < 0.05; Figure 7B), oAECs cultured in Petri dishes, as expected, had always a significantly higher PI respect to all PLGA samples (*p* < 0.05; Figure 7B,C).

The obtained results allow to hypothesize that the increased cellularity on CAP-treated PLGA microfibers could be attributed to cell adhesion rather than cell proliferation.

**Figure 8:**
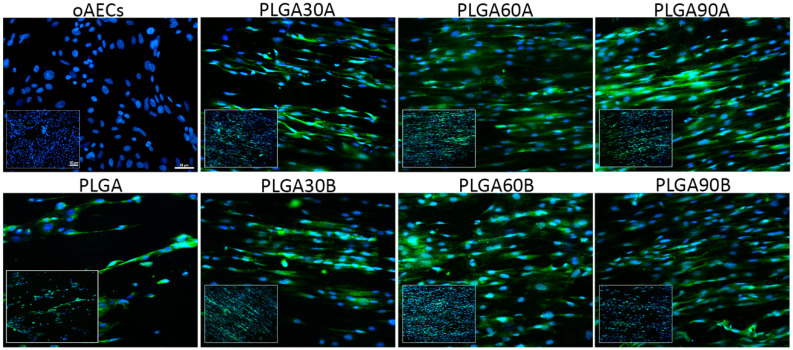
Teno-inductive properties of untreated and CAP treated PLGA microfibers on AECs. Immunocytochemical analysis revealed TNMD protein expression (green fluorescence) in oAECs seeded onto untreated and CAP treated PLGA microfibers after 24 h; whereas cells cultured on Petri dishes do not express this protein. DAPI (blue fluorescence) counterstained nuclei. Positivity to TNMD was evident in the cytoplasm of the oAECs. Scale bars = 20 μm. Insets show the same images at a lower magnification; scale bars = 50 µm; (*n* = 3 for each type of sample/ time point, sample size: 15 mm × 7 mm).

It was finally verified if the CAP treatment could maintain the early teno-inductive potential of the aligned PLGA microfibers on oAECs without adding tenogenic supplementation to the culture media. AECs’ tenogenic differentiation on PLGA microfibers was confirmed by analyzing TNMD protein expression, one of the most recognized tendon related markers, by using the immunofluorescence technique on oAECs seeded on Petri dishes, and onto untreated and treated CAP PLGA microfibers. The oAECs do not normally express TNMD protein in their cytoplasm [90], and as shown in Figure 8, the cells were still negative to this protein when cultured on Petri dishes during all culture times. Instead, TNMD protein was already expressed after only 24 h when cultured onto all PLGA microfiber groups (Figure 8) and positivity was also maintained after 48 h culture (data not shown), demonstrating that CAP treatment maintained the teno-inductive potential of PLGA microfibers.

The obtained results confirm that PLGA is biocompatible with oAECs [10,25,64] and that the enhanced hydrophilic properties that gave to the electrospun PLGA microfibers, has improved cell adhesion and penetration. It must be noticed that the best cell infiltration within the CAP treated PLGA microfibers was obtained when the PLGA microfibers were CAP treated from the lower working distance (PLGA30A, PLGA60A and PLGA90A). Moreover, better effects for the cell adhesion were obtained on PLGA60A respect to the all other groups.

Indeed, it can be assumed that the better cell adhesion and infiltration could be explained by the fact that lowering the treatment working distance results in a higher oxygen content that may favor cell-material interaction. These results are in agreement with other literature data reporting the improved cell adhesion and proliferation on electrospun scaffolds made of different biomaterials and treated with different types of plasma [51,67,109,110,111,112,113]. The enhanced cell performance could be attributed to the increased plasma-induced hydrophilicity that could allow the adsorption of more proteins secreted by the cultivated cells without altering their natural conformation on the surface of treated PLGA microfibers [114]. Consequently, more cellular receptors can bind to the adsorbed proteins leading to numerous focal adhesive sites enhancing cell adhesion [93]. In-deep analysis elucidates that the introduced oxygen-containing functionalities, responsible for the increased wettability, are specifically correlated with the enhanced cell adherence and penetration. Carboxyl, carbonyl and hydroxyl groups, that firmly bind proteins, could recruit more integrins and act as a glue strongly connecting and stabilizing the anchor points of focal adhesive complexes [113,115,116]. It has been also described an improved cell migration into the scaffold′s depth [55]. In-vitro cell infiltration studies showed that plasma treatments effectively enhance cell migration into the microfibrous scaffolds [55], as also in in-vivo experiments involving the subcutaneous implantation of plasma-treated PLLA scaffolds under the skin of Sprague-Dawley rats also showed increased cell infiltration [57].

Although, differently to the cited papers, in which it was observed that cells seeded on CAP treated materials with a random fiber pattern had an enhanced cell proliferation, in this research, cell proliferation and teno-differentiation were not influenced by CAP treatment. The reduced PI observed respect to the oAECs cultured on Petri dishes could be a consequence of oAECs’ pre-commitment towards the tenogenic lineage when cultured on highly aligned PLGA microfibers. It is reasonable that the rapid teno-differentiation of the oAECs have stopped their proliferation as already demonstrated in previous works [10,25]. In this research, thus, it has been confirmed that the intrinsic physical cues of the tendon mimetic aligned PLGA fleeces are able to boost oAECs tenogenic differentiation. In fact, in previous works, it was also demonstrated that not only fiber topography [10], but also fiber diameter size [25] had a great influence on the teno-differentiation ability of oAECs. In Russo et al. [10], oAECs cultured on the PLGA fleeces with both highly aligned and randomly oriented fibers up to 28 days, have shown an upregulation of *COL1* and *TNMD* mRNAs and COL1 protein already after 48 h of culture on the cells seeded onto PLGA microfibers with aligned topography, while in El Khatib et al. [25], it was observed an upregulation of *SCX*, *COL1* and *TNMD* mRNAs and COL1 protein in oAECs cultured onto PLGA fleeces with aligned microfibero possessing two different fiber diameter sizes, already after 24 h of culture. In this research, TNMD protein expression was already evident in the cytoplasm of oAECs seeded onto both on untreated and treated CAP PLGA microfibers after 24 h of culture. The obtained results are in accordance with other works that confirm the influence of fiber alignment and diameter on tenogenic differentiation of MSCs occurred after at least 3 days of culture [11,12,27,117,118]. However, to our knowledge, it has been demonstrated for the first time that CAP treatment did not affect cell tenogenic differentiation confirming the maintenance of PLGA inductive properties even after being plasma treated. CAP treatment influenced PLGA microfiber hydrophilicity and not its topographical architecture and bulk structure as demonstrated by the SEM, molecular weight determination and FTIR analysis. Indeed, only few researches conducted in other models have demonstrated that CAP treatments do not affect cell differentiation towards on cartilage [49,119], osteo [52,120,121], hepatic [122] or neuronal [60] lineages. Thus, both fiber alignment and diameter size regulate oAECs proliferation and teno-differentiation, whereas cell adhesion and penetration within the microfibers are influenced by CAP treatment. The present study highlights the synergistic effect of PLGA fiber size, orientation and surface chemistry on the bioresponsive performance of oAECs.

Surface modification techniques, in particular CAP, have the ability to activate the rather less-bioactive polymers and allow the incorporation of bioactive agents on their surfaces rendering in turn these materials more biofunctional for more specific applications [45,54,123,124]. Future goals could be based on the functionalization of these activated PLGA microfibers with cocktail of growth factors/bioactive molecules to support oAECs tenogenic differentiation and hence improve tendon regeneration.

## 4. Conclusions

In this research it has been explored the effect of surface chemistry modification of electrospun PLGA microfibers with aligned microfibers via CAP on oAECs performance. Indeed, CAP treatment improved cell adhesion and penetration within the CAP treated PLGA microfibers characterized by the increased hydrophilicity due to the increase of oxygen content while maintaining PLGA biocompatibility and teno-inductive properties for oAECs. In particular, since the treated PLGA microfibers are addressed for tendon tissue engineering applications, the optimal set of conditions for plasma treatment were defined based on high cell adhesion and penetration as well as best mechanical properties of the PLGA microfibers. Based on these premises, CAP treatment conducted on electrospun PLGA microfibers for 60 s from a working distance of 1.3 cm (PLGA60A) is considered as the optimum surface treatment conditions since it conjugates a balance of hydrophilicity, high oAECs’ adhesion and penetration, mechanical properties and maintaining at the same time the teno-inductivity potential.

Overall, non-thermal plasma technology represents a very promising technique for surface modification of electrospun fibrous matrices to improve their hydrophilic properties and hence cell performance for tissue engineering applications. In addition, carboxyl, carbonyl and hydroxyl groups functionalities obtained through the atmospheric plasma approach, could be in the future a useful platform for additional conjugation reactions with bioactive molecules and drugs, also in view of regenerative medicine and drug delivery purposes.

## Figures and Tables

**Table 1:** Annotations of different plasma treated PLGA sample types.

Parameters	Plasma Treated PLGA Sample Types					
	PLGA30A	PLGA30B	PLGA60A	PLGA60B	PLGA90A	PLGA90B
Exposure time	30 s	30 s	60 s	60 s	90 s	90 s
Distance	1.3 cm	1.7 cm	1.3 cm	1.7 cm	1.3 cm	1.7 cm

**Table 2:** Fiber alignment of the untreated and CAP treated PLGA microfibers represented by direction, dispersion and goodness.

Sample	Fiber Diameter (µm)	Direction (°)	Dispersion (°)	Goodness
PLGA	1.366 ± 0.14	−9.98	3.55 ± 0.14	0.99
PLGA-30A	1.355 ± 0.12	23.14	3.61 ± 0.23	1
PLGA-30B	1.361 ± 0.10	−39.42	3.55 ± 0.31	0.99
PLGA-60A	1.375 ± 0.10	14.43	3.72 ± 0.21	0.98
PLGA-60B	1.381 ± 0.13	0.48	4.30 ± 0.17	0.98
PLGA-90A	1.387 ± 0.11	10.81	4.01 ± 0.21	0.98
PLGA-90B	1.389 ± 0.12	−76.52	3.71 ± 0.23	0.94
